# COX-2-mediated stimulation of the lymphangiogenic factor VEGF-C in human breast cancer

**DOI:** 10.1038/sj.bjc.6603067

**Published:** 2006-03-28

**Authors:** A V Timoshenko, C Chakraborty, G F Wagner, P K Lala

**Affiliations:** 1 Department of Anatomy and Cell Biology, The University of Western Ontario, London, Ontario, Canada N6A5C1; 2 Department of Biology, The University of Western Ontario, London, Ontario, Canada N6A5B7; 3 Department of Pathology, The University of Western Ontario, London, Ontario, Canada N6A5C1; 4 Department of Physiology and Pharmacology, The University of Western Ontario, London, Ontario, Canada N6A5C1

**Keywords:** breast cancer, VEGF-C, COX-2, EP receptors, lymphangiogenesis

## Abstract

Increased expression of COX-2 or VEGF-C has been correlated with progressive disease in certain cancers. Present study utilized several human breast cancer cell lines (MCF-7, T-47D, Hs578T and MDA-MB-231, varying in COX-2 expression) as well as 10 human breast cancer specimens to examine the roles of COX-2 and prostaglandin E (EP) receptors in VEGF-C expression or secretion, and the relationship of COX-2 or VEGF-C expression to lymphangiogenesis. We found a strong correlation between COX-2 mRNA expression and VEGF-C expression or secretion levels in breast cancer cell lines and VEGF-C expression in breast cancer tissues. Expression of LYVE-1, a selective marker for lymphatic endothelium, was also positively correlated with COX-2 or VEGF-C expression in breast cancer tissues. Inhibition of VEGF-C expression and secretion in the presence of COX-1/2 or COX-2 inhibitors or following downregulation of COX-2 with COX-2 siRNA established a stimulatory role COX-2 in VEGF-C synthesis by breast cancer cells. EP1 as well as EP4 receptor antagonists inhibited VEGF-C production indicating the roles of EP1 and EP4 in VEGF-C upregulation by endogenous PGE_2_. Finally, VEGF-C secretion by MDA-MB-231 cells was inhibited in the presence of kinase inhibitors for Her-2/neu, Src and p38 MAPK, indicating a requirement of these kinases for VEGF-C synthesis. These results, for the first time, demonstrate a regulatory role of COX-2 in VEGF-C synthesis (and thereby lymphangiogenesis) in human breast cancer, which is mediated at least in part by EP1/EP4 receptors.

Overexpression of cyclooxygenase (COX)-2 is now recognized as a marker for tumour progression documented for cancers of the colon (Soslow *et al*, 2000), lung (Hida *et al*, 1998; Soslow *et al*, 2000), head and neck (Chan *et al*, 1999), pancreas (Tucker *et al*, 1999) and the breast (Parrett *et al*, 1997; Soslow *et al*, 2000). A functional role of COX-2 in tumour development and progression has been demonstrated by both overexpression (Liu *et al*, 2001) and disruption (Chulada *et al*, 2000) of the COX-2 gene as well as application of drugs blocking both COX-1/-2 or COX-2 alone (Lala *et al*, 1997; Harris, 2003; Wang and DuBois, 2004). This role has primarily been attributed to elevated levels of prostanoids, mainly prostaglandin E_2_ (PGE_2_), in the tumour microenvironment (Rolland *et al*, 1980). We had earlier demonstrated that tumour-derived PGE_2_ acts as a paracrine as well as an autocrine factor to promote breast cancer progression and metastasis by multiple mechanisms, namely by inactivation of host anti tumour immune cells and a stimulation of tumour cell migration, invasiveness and tumour-associated angiogenesis (Lala and Saarloos, 1994; Lala *et al*, 1997; Rozic *et al*, 2001).

PGE_2_ action depends on activation of one or more of the four PGE_2_ receptors (EP1-EP4) expressed by target cells. They are encoded by different genes and coupled with different G-proteins: EP1 coupled with Gq, EP2 and EP4 coupled with Gs, and certain transcripts of EP3 coupled with Gi (Breyer *et al*, 2001). Role(s) of specific EP receptor-mediated signalling in tumour development and progression has so far been shown to vary with the tumour model and the specific cellular functions contributing to the metastatic phenotype of cancer cells. For example, EP1, EP2 and EP4 contributed to colon carcinogenesis (Hull *et al*, 2004), and EP2 was shown to be required for COX-2-mediated mammary hyperplasia (Chang *et al*, 2005). Furthermore, EP4 contributed to the stimulation of migration of colorectal (Sheng *et al*, 2001) and breast (Timoshenko *et al*, 2003) cancer cells. EP4 receptors were also responsible for an upregulation of iNOS gene expression under inducible conditions in murine breast cancer cells that increased their invasive capacity (Timoshenko *et al*, 2004), as well as in osteoclast development and bone metastasis in a breast cancer model (Ohshiba *et al*, 2003).

Whereas the role of COX-2 in promoting tumour-associated angiogenesis is well-documented (Tsujii *et al*, 1998), possible role of COX-2 in lymphangiogenesis and lymphatic metastasis remains poorly defined. Two members of the vascular endothelial growth factor (VEGF) family that is, VEGF-C and VEGF-D have been shown to promote lymphangiogenesis by binding to VEGF receptor VEGFR-3 on lymphatic endothelial cells (Saharinen *et al*, 2004). Forced VEGF-C overexpression in a VEGF-C-nonexpessing and nonmetastatic human breast cancer cell line MCF-7 resulted in enhanced tumour growth *in vivo*, lymphangiogenesis and lymphatic metastasis in immunodeficient mice (Mattila *et al*, 2002). Elevated expression of VEGF-C in tumour tissues has been shown to have a negative influence on prognosis and a positive correlation with lymph node metastasis in many cancers including cancers of the breast (Nakamura *et al*, 2003), uterine cervix (Fujimoto *et al*, 2004), colon and rectum (Onogawa *et al*, 2004), oesophagus (Kimura *et al*, 2003), stomach (Duff *et al*, 2003a), head and neck (O-charoenrat *et al*, 2001), and gallbladder (Nakashima *et al*, 2003). Additionally, serum VEGF-C was shown to be elevated in patients with non-small cell lung cancer (Tamura and Ohta, 2003) and colorectal cancer (Duff *et al*, 2003b), and in the former case, this was also correlated with lymph node metastasis. Interestingly, a positive association between COX-2 and VEGF-C mRNA expression has been reported in oesophageal adenocarcinoma (von Rahden *et al*, 2005). A similar association between COX-2 and VEGF-C was also demonstrated at the protein levels by immunohistochemical studies of squamous cell carcinomas of the head and neck (Kyzas *et al*, 2005) and oesophagus (Byeon *et al*, 2004) as well as in non-small cell lung adenocarcinoma (Su *et al*, 2004). A role of COX-2 in VEGF-C upregulation was suggested in the case of non-small cell lung cancer cells (Su *et al*, 2004) as well as oesophageal adenocarcinoma cells (von Rahden *et al*, 2005). To date, however, no information exists regarding whether COX-2 is causally associated with VEGF-C upregulation and thereby lyphangiogenesis in breast cancer, and if so, what is the role of EP receptors on cancer cells in this event.

Whether cancer metastasis to lymph nodes depends on pre-existing or newly formed lymphatics still remains a debated issue. Intratumoral lymphangiogenesis, as identified by the lymphatic endothelium-specific marker LYVE-1 (Saharinen *et al*, 2004), is a salient feature of invasive head and neck cancer (Beasley *et al*, 2002) and also inflammatory breast cancer (Van der Auwera *et al*, 2004). However, this is not so for noninflammatory, invasive human breast carcinomas (Williams *et al*, 2003; Vleugel *et al*, 2004; Van der Auwera *et al*, 2004). It the latter case, lymphatic vessels were demonstrated only in the peritumoral region (Vleugel *et al*, 2004) and it is unclear whether they represent pre-existing or newly formed lymphatics. Thus a paracrine role of breast cancer-derived VEGF-C in lymphangiogenesis and lymphatic metastasis remains an open question.

Present study utilized several well-established human breast cancer cell lines varying in COX-2 expression and metastatic abilities as well as numerous human breast cancer specimens with the following objectives: (1) to examine the relationship between COX-2 (mRNA) expression and VEGF-C (mRNA) expression (cell lines and tissues) or VEGF-C secretion (cell lines); (2) to examine the relationship between COX-2 or VEGF-C expression and the expression of LYVE-1, a marker for the lymphatic endothelium, in breast cancer tissues; (3) to examine the causal relationship between COX-2 activity or gene expression and VEGF-C expression/secretion in high COX-2 and VEGF-C expressing cell lines; (4) to identify the role (s) of specific EP receptors in endogenous PGE_2_-mediated VEGF-C stimulation in these cells.

## MATERIALS AND METHODS

### Reagents

PGE_2_, 17-phenyl trinor PGE_2_ (EP1 agonist) and SC-560 (selective COX-1 inhibitor) were purchased from Cayman Chemicals (Ann Arbor, MI, USA). NS-398 (selective COX-2 inhibitor), PP1 (Src kinase inhibitor) and SC-51322 (EP1 antagonist) were from Biomol (Plymouth Meeting, PA, USA). AH-23848B (EP4 antagonist) was from Glaxo/Wellcome (Stevenage, UK). Concanavalin A (Con A), indomethacin (nonselective COX-1/COX-2 inhibitor), and 3,3′-diaminobenzidine tablets were from Sigma (Oakville, ON, Canada). PD153035 (Her2/neu kinase inhibitor) and SB203580 (p38 kinase inhibitor) were from Calbiochem (San Diego, CA, USA). L-161982 (EP4 antagonist) was kindly provided by Dr M Young from Merck Frosst (Kirkland, QC, Canada).

### Human breast cancer cell lines

Human breast cancer cell lines MCF-7, T-47D, Hs578T and MDA-MB-231 were obtained from the American Type Culture Collection and grown in DMEM (Invitrogen/GIBCO, Burlington, ON, Canada) supplemented with 8% FBS, 25 mM HEPES buffer, 50 U ml^−1^ penicillin and 50 *μ*g ml^−1^ streptomycin. Propagation of MCF-7 and T-47D cells were performed in the presence of 0.01 mg ml^−1^ bovine insulin.

### Human breast cancer tissues

Frozen tissue samples of 10 surgically resected human breast cancer specimens were obtained from The London Health Sciences Center, London Laboratory Services Group, London, Ontario without any preselection. The study was approved by the Tissue and Archives Committee, Department of Pathology, the University of Western Ontario. Histological data were available on eight out 10 specimens. Of these, two were lymph node positive and six had no demonstrable metastasis in the resected lymph nodes. The tumours represented infiltrating, invasive ductal, lobular, or ducto-lobular carcinomas of various SBR grades (I–III). None was described as inflammatory breast cancer.

### RT–PCR

First-strand cDNAs were synthesized from 2 *μ*g of TRIzol reagent-extracted total RNA from breast cancer cells and lesions using the SuperScript™ II Reverse Transcriptase (Invitrogen, Burlington, ON, Canada). Regular hot start (2 min, 94°C) PCR was performed in a 20 *μ*l volume containing 18 *μ*l Platinum® PCR SuperMix (Invitrogen), 0.8 *μ*l template cDNA solution, and 0.8 *μ*l primer mixture (25 pmol *μ*l^−1^ each). PCR was run for 30–35 cycles of denaturation 94°C (30 s), annealing 55°C (30 s), extension 72°C (45 s) followed by 5 min of final extension at 72°C. Primers for VEGF family, COX-2, LYVE-1 and GAPDH (Table 1) were synthesized locally at the UWO Oligo Factory (London, Canada) or ordered from Sigma/Genosys (Oakville, ON, Canada). All primers were designed and evaluated using Oligo Explorer and Oligo Analyzer software (Teemu Kuulasmaa, Finland) except for LYVE-1, VEGF-A and VEGF-D pairs (41–43). PCR products were separated on 1% agarose gel containing 0.25 *μ*g ml^−1^ ethidium bromide and visualized under UV light. Real-time quantitative PCR (qPCR) for VEGF-C, COX-2, LYVE-1 and GAPDH was performed in single microcapillary tubes using the LightCycler™ (Roche Diagnostic, Laval, Canada) and SYBR® Green Tag ReadyMix™ (Sigma, St Louis, USA). Cycling parameters were optimized as follow: denaturation 94°C (0 s), annealing 55°C (5 s), extension 72°C (24 s) and detection 80°C (1 s). Each microcapillary contained 7.1 *μ*l nuclease free H_2_O, 10 *μ*l SYBR reagent, 0.5 *μ*l template cDNA, 1.6 *μ*l 25 mM MgCl_2_ and 0.8 *μ*l 25 pmol *μ*l^−1^ primer mixture. The cycler software was used for quantification of COX-2, VEGF-C and LYVE-1 mRNA levels relative to GAPDH mRNA expression.

### ELISA for VEGF-A, VEGF-C and VEGF-D

The levels of VEGF-A and VEGF-C accumulating in serum-free cell culture media were measured using Human VEGF and VEGF-C EIA kits from Immuno-Biological Laboratories (Gumna, Japan). The levels of VEGF-D were measured using Quantikine® Human VEGF-D Immunoassay kit from R&D (Minneapolis, MN, USA).

### COX-2 siRNA transfection

The Silencer siRNA Transfection Kit and predesigned siRNA from Ambion (St Austin, TX, USA) were used to transfect MDA-MB-231 cells with COX-2 siRNA by neofection method. The conditions of neofection were optimized by using GAPDH siRNA as a positive control and siPORT NeoFX was selected as the most efficient transfection agent. To perform transfection with COX-2 siRNA, 2.3 ml of cells (0.1 × 10^6^ cells ml^−1^) in complete DMEM were mixed with 200 *μ*l of the transfection complex containing 125 nM of siRNA and 2% of siPORT NeoFX in OPTI-MEM I medium and added to a well of six-well culture plate. The cells were cultured for 48 h at 37°C and the efficiency of transfection was assayed by qPCR. To analyse VEGF-C secretion, the monolayer of COX-2 siRNA-treated cells was rinsed with serum-free DMEM and incubated for additional 24 h in the serum-free medium (2 ml well^−1^).

### Immunohistochemistry for VEGF-C

MDA-MB-231 cells were grown up to subconfluency on Lab-Tek Permanox slides with four chambers from Nalge Nunc (Naperville, IL, USA). The complete medium was replaced by serum-free DMEM and the cells were preincubated for 1 h with serum-free DMEM followed by 24 h incubation with or without inhibitors as specified in the results. The cell monolayers were rinsed with PBS, fixed in 2% formaldehyde for 30 min, washed again two times with PBS, and treated for 5 min with 2% glycine. The slides were then immunostained using anti-human VEGF-C rabbit IgG from IBL (Gunma, Japan), the Vestatin Elite ABC kit from Vector Laboratories (Burlingame, CA, USA) and 3,3′-diaminobenzidine for colour development according to the manufacturers'protocol.

### MTT assay for cell proliferation/survival

All agents (pharmacological agents, inhibitors), the effects of which were tested on VEGF-C production by human breast cancer cells were also tested under identical conditions for possible effects on cell proliferation/survival using the MTT Cell Proliferation Kit I from Roche Diagnostics (Laval, QC, Canada), as reported earlier (Timoshenko *et al*, 2003).

### Statistics

All mean values and standard deviations (s.d.) from at least triplicate (often quadruplicate) measurements were calculated with Microsoft Office Excel 2003 (Microsoft Corporation, Seattle, WA, USA). Statistical significance of differences between two groups was determined with a two-sided Student's *t*-test considering *P*<0.05 as an indicator of significant difference between means. Correlations between levels of COX-2, VEGF-C and LYVE-1 mRNA levels were made with nonparametric Spearman correlation test providing correlation coefficients and *P*-values. Linear regression analysis of the data including 95% confidence intervals was performed with statistical package of GraphPad Prism (GraphPad Software Inc., San Diego, CA, USA).

## RESULTS

### High VEGF-C expression and production distinguishes a highly metastatic from a nonmetastatic breast cancer cell line

**Figure 1:**
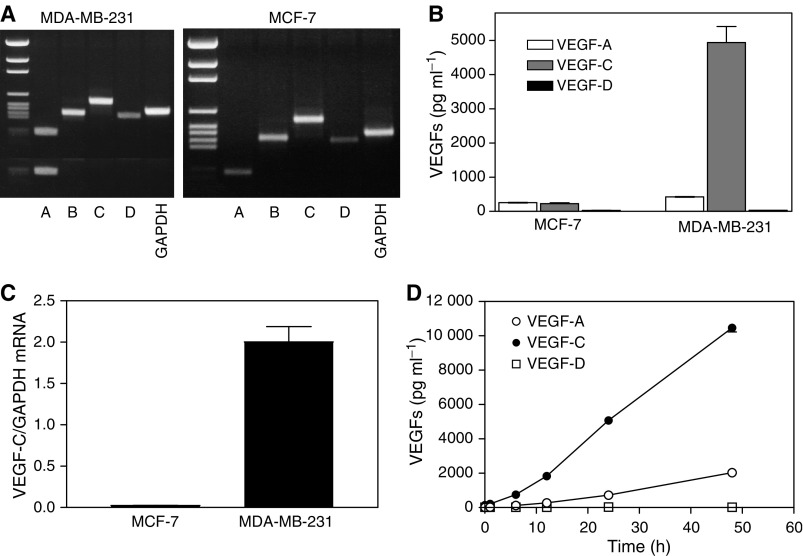
Expression of VEGF-A, -B, -C and -D mRNAs and VEGF-A, -C, and –D protein secretion by human breast cancer cell lines MDA-MB-231 and MCF-7. (**A**) RT–PCR-based amplification of VEGF mRNAs, showing that message for each VEGF class is detectable in both cell lines. (**B**) Accumulation of secreted VEGF-A, -C and -D proteins in serum-free culture media of both cell lines at 24 h showing high VEGF-C production by MDA-MB-231 cells. (**C**) Differences in expression of VEGF-C mRNA by MDA-MB-231 and MCF-7 cells as revealed by real time qPCR. (**D**) Kinetics of accumulation of VEGF-A, -C and -D protein in culture media of MDA-MB-231 cells. Data in (**B**, **C**, and **D**) represent mean±s.d. (*n*=4).

Highly metastatic MDA-MB-231 and nonmetastatic MCF-7 human breast cancer cell lines were found to express the mRNA, although at different levels, for all four VEGFs (A, B, C, D) as detected by RT–PCR (Figure 1A). Both cell lines secreted immunodetectable levels of VEGF-A and VEGF-C but very little VEGF-D at 24 h (Figure 1B). MCF-7 cells produced low levels of VEGF-C as well as VEGF-A. In comparison with MCF-7 cells, VEGF-A production by MDA-MB-231 cells was two-fold higher, whereas VEGF-C production was about 25-fold higher (Figure 1B). These findings are consistent with overexpression of VEGF-C mRNA in MDA-MB-231 cells as opposed to low expression in MCF-7 cells, as measured by real-time qPCR (Figure 1C). Accumulation of both VEGF-A and VEGF-C in serum-free culture media of MDA-MB-231 cells increased linearly for at least 48 h and, again, no detectable level of VEGF-D was noted at any time point (Figure 1D). The rate of VEGF-C production by these cells was about 10-fold higher than that of VEGF-A. Thus, the elevated expression and production of the lymphangiogenic factor VEGF-C seems to be an important feature of this highly metastatic breast cancer cell line.

### VEGF-C production by human breast cancer cells and VEGF-C expression by human breast cancer tissues correlate positively with COX-2 expression

As high levels of COX-2 expressed by MDA-MB-231 cells was shown to contribute to its metastatic phenotype whereas nonmetastatic MCF-7 cells did not express COX-2 (Liu and Rose, 1996), we examined whether there is a relationship between COX-2 expression and VEGF-C producing ability, using additional human breast cancer cell lines T-47D (nonmetastatic) and Hs578T (metastatic).

**Figure 2:**
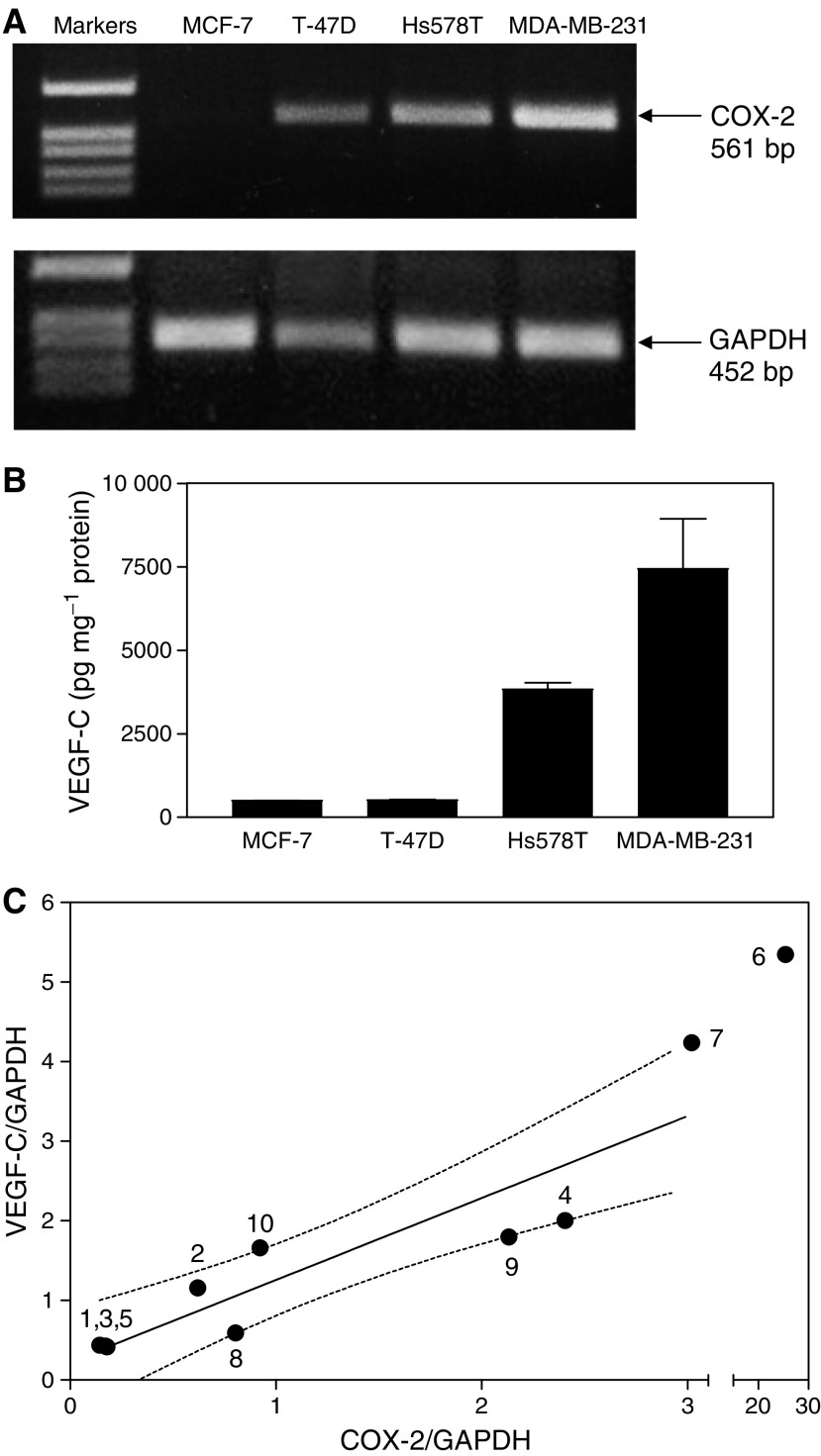
Relationship between COX-2 expression and VEGF-C synthesis/expression in human breast cancer cell lines and tissues. (**A**) Expression of COX-2 and GAPDH genes in MCF-7, T-47D, Hs578T and MDA-MB-231 human breast cancer cells as detected by RT–PCR. COX-2 expression is not detectable in MCF-7, weak in T-47D, moderate in Hs578T, and high in MDA-MB-231 cells. (**B**) Accumulation of VEGF-C in culture media of the above breast cancer cell lines at a 24 h correlates with their levels of COX-2 expression. Data represent mean±s.d. (*n*=4). (**C**) Correlation between relative levels of COX-2 mRNA and VEGF-C mRNA (standardized against GAPDH mRNA) expression in human breast cancer tissues (*n*=10) measured with real-time qPCR reveals a strong positive association between the two parameters (*r*=0.94, *P*=0.0002). Numbers on the graph present patient identifiers, and dotted lines present 95% confidence intervals of the regression line.

As detected by RT–PCR, all the four human breast cancer cell lines tested expressed similar levels of COX-1 mRNA but significantly different levels of COX-2 mRNA. Thus, MCF-7 cells were COX-2 negative, T-47D expressed very low levels of COX-2, Hs578T expressed moderately high levels, whereas MDA-MB-231 expressed very high levels of COX-2 (Figure 2A). The levels of VEGF-C secretion by these cell lines at 24 h (Figure 2B) clearly correlated with their relative COX-2 expression levels.

To find out whether the observed positive correlation between COX-2 and VEGF-C expression or production by breast cancer cell lines reflect a similar relationship *in vivo*, we analysed mRNA levels for both genes in human breast cancer tissue samples from 10 randomly selected surgically removed specimens. The results of the real-time qPCR study showed that, indeed, there was a strong positive association (*r*=0.94, *P*=0.0002) between COX-2 and VEGF-C mRNA levels (Figure 2C). Taken together, these data clearly demonstrated a positive association between COX-2 and VEGF-C systems in breast cancer cells *in vitro* as well as *in vivo*.

### LYVE-1 expression in human breast cancer tissues is positively correlated with COX-2 and VEGF-C expression

**Figure 3:**
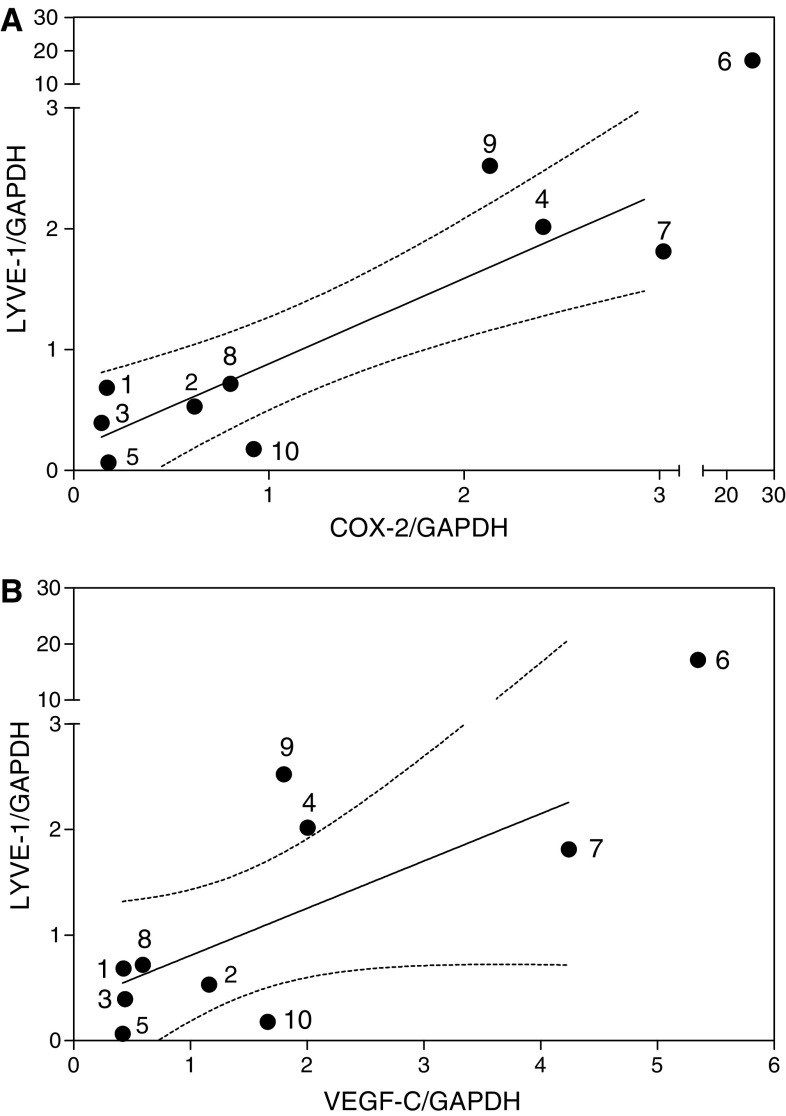
Relationship between LYVE-1 and COX-2 (**A**) or VEGF-C (**B**) mRNA expression in the same breast cancer tissues (*n*=10) used for data in Figure 2C. A positive association (*r*=0.75, *P*=0.017 and *r*=0.78, *P*=0.013, respectively) is revealed in both cases; dotted lines present 95% confidence interval of the regression line.

LYVE-1 is a highly selective marker of lymphatic endothelial cells (Saharinen *et al*, 2004) and is not expressed by MDA-MB-231 breast cancer cells (Cunnick *et al*, 2001). The level of LYVE-1 mRNA expression in tumour samples was shown to be a sensitive indicator of the level of lymphangiogenesis *in vivo* (Cunnick *et al*, 2001; Van der Auwera *et al*, 2004). For this reason, we analysed mRNA level of the LYVE-1 gene in the same breast cancer tissue samples in which COX-2 and VEGF-C mRNA levels were measured. The results of the real time qPCR revealed a robust correlation of LYVE-1 mRNA levels in these tissue samples with COX-2 (*r*=0.75, *P*=0.017) as well as VEGF-C (*r*=0.78, *P*=0.013) expression levels (Figure 3A and B).

### COX-2 activity and VEGF-C expression/production are causally related

**Figure 4:**
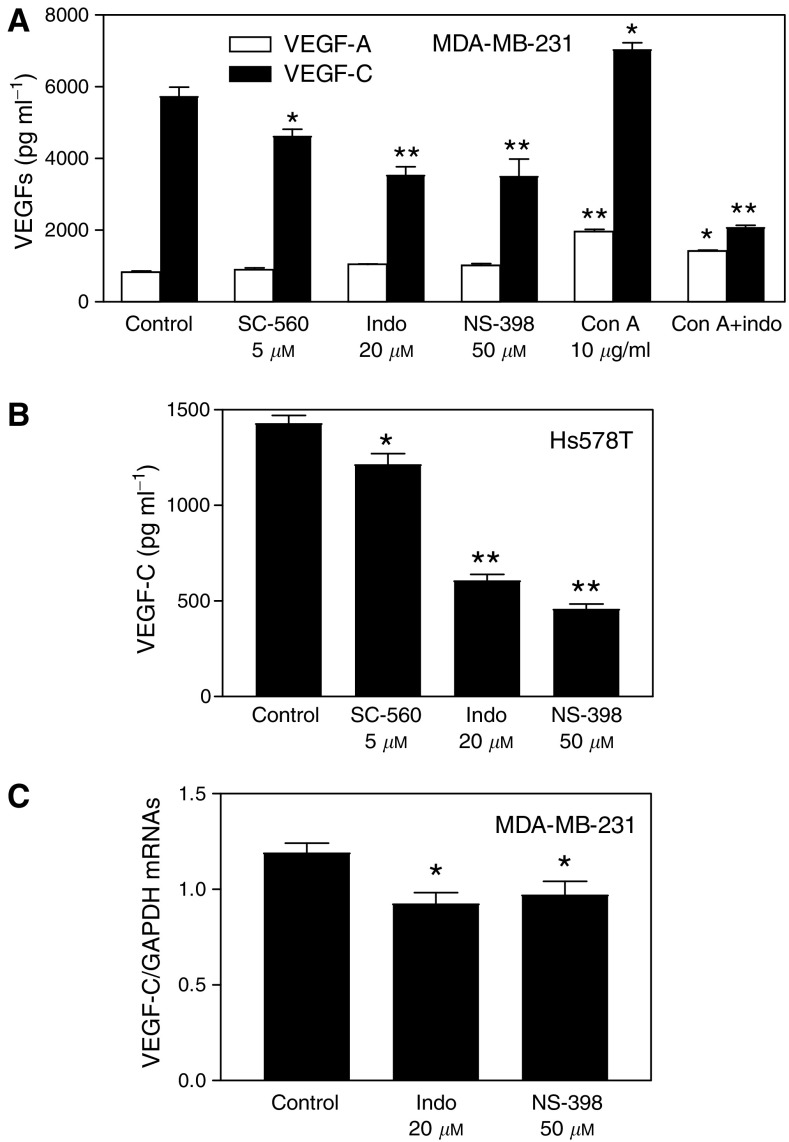
Effects of COX inhibitors on VEGF-C and VEGF-A secretion and VEGF-C expression in breast cancer cells. (**A**) Effects of COX-1 inhibitor SC-560, COX-1/-2 inhibitor indomethacin, and COX-2 inhibitor NS-398 on VEGF-C or VEGF-A production by COX-2 expressing MDA-MB-231 cells. (**B**) Effects of same inhibitors on VEGF-C production by COX-2 expressing Hs578T cells. COX-1 inhibitor caused a minor (but significant; *P*<0.05) reduction, whereas COX-2 or COX-1/-2 inhibitors caused a substantial (*P*<0.01) and similar reduction in VEGF-C production by both cell lines. However, these inhibitors have no inhibitory effect on VEGF-A production. Con A, known to stimulate PGE_2_ production by MDA-MB-231 cells, also stimulated VEGF-C as well as VEGF-A production, which were substantially (*P*<0.01) blocked with COX-1/2 inhibitor. (**C**) Effect of 24 h treatment with COX inhibitors (20 *μ*M indomethacin or 50 *μ*M NS-398 in serum-free culture) on VEGF-C mRNA expression in MDA-MB-231 cells. Data represent mean±s.d. (*n*=4). ^*^P<0.05 and ^**^P<0.001 in comparison to control untreated cells.

To examine whether COX-2 activity played any role in regulating VEGF-C synthesis by human breast cancer cells, we tested the effects of COX-1/-2 inhibitors (indomethacin, NS-398 and SC-560) on VEGF-C accumulation in cell culture media. For a comparison, VEGF-A production was measured side by side. The inhibitors were added at concentrations which were nontoxic (having no effects on cell proliferation/survival) for cells as detected by MTT assay (data not shown). We found that the nonselective COX-1/-2 inhibitor indomethacin (20 *μ*M) as well as the selective COX-2 inhibitor NS-398 (50 *μ*M) strongly suppressed but did not abrogate VEGF-C production by both COX-2 expressing cell lines MDA-MB-231 (Figure 4A) and Hs578T (Figure 4B). Similar inhibitory effects of COX-1/-2 and COX-2 inhibitors implicate the regulatory role of COX-2 on VEGF-C production. In contrast, VEGF-A production by MDA-MB-231 cells was not reduced in the presence of these inhibitors (Figure 4A). We had earlier shown that these concentrations of indomethacin and NS-398, respectively, suppressed PGE_2_ production by 72 and 94% in MDA-MB-231 cells (Timoshenko *et al*, 2003). Concanavalin A (Con A), earlier shown to stimulate PGE_2_ production by MDA-MB-231 cells (Timoshenko *et al*, 2003), also stimulated VEGF-C as well as VEGF-A production, which were significantly blocked with the COX-1/2 inhibitor (Figure 4A). This inhibition was much higher in the case of VEGF-C. A small inhibitory effect on VEGF-C but not VEGF-A was also observed with the selective COX-1 inhibitor SC-560 (5 *μ*M) (Figure 4A and B) but it was significantly less than that caused by the selective COX-2 inhibitor NS-398 (Figure 4A). In line with these findings, NS-398 and indomethacin were also found to downregulate VEGF-C mRNA expression in MDA-MB-231 cells validating a role of COX-2 as an upstream regulatory enzyme (Figure 4C). These results, taken together, reveal that VEGF-C synthesis by breast cancer cells is, at least in part, upregulated by endogenous COX-2 activity.

### Knock down of COX-2 mRNA reduces VEGF-C production

**Figure 5:**
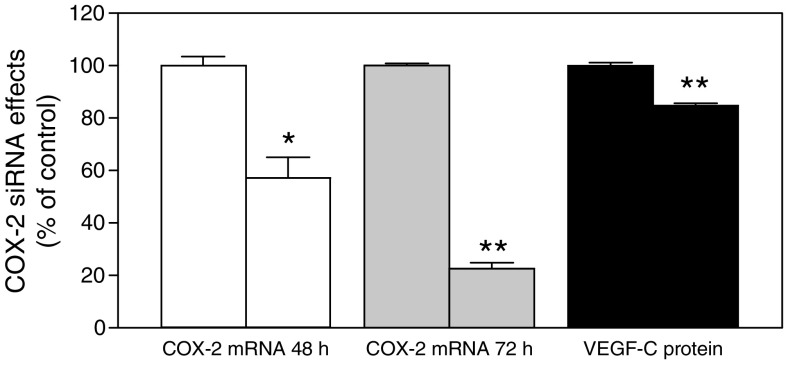
Effects of COX-2 siRNA treatment on COX-2 expression and VEGF-C production by MDA-MB-231 cells. The first bar in each series is control, that is, the treatment with scrambled siRNA, and the second bar is experimental, that is, the treatment with COX-2 siRNA. siRNA treatment was conducted in serum containing medium for 48 h. Cells were washed and cultured in serum free media for another 24 h to measure VEGF-C protein accumulation in the cell free media, and COX-2 mRNA levels in cells at both 48 and 72 h following the siRNA treatment. A significant suppression of COX-2 mRNA expression as well as VEGF-C secretion was noted in COX-2 siRNA treated cells. ^*^P<0.01, ^**^P<0.001.

To examine whether the COX-2 gene plays a regulatory role in VEGF-C synthesis by human breast cancer cells, we adopted the siRNA approach to knock down COX-2 gene in high COX-2 expressing MDA-MB-231 cells. As shown in Figure 5, COX-2 siRNA-treated cells exhibited a significant reduction in both COX-2 mRNA expression as well as VEGF-C production. However, the level of reduction in VEGF-C production by cells during a 24 h period following COX-2 siRNA pretreatment for 48 h was relatively less then the levels of reduction in COX-2 mRNA expression either at 48 or 72 h. These data may indicate that the presence of additional gene (s) other than COX-2 regulating VEGF-C synthesis in MDA-MB-231 cells.

### EP1 and EP4 receptors contribute to VEGF-C production by highly metastatic human breast cancer cells

We had earlier shown that PGE_2_ is the major prostanoid resulting from COX-2 expression in highly metastatic breast cancer cells of different origin and that MDA-MB-231 cells express mRNA for each of the four PGE_2_ receptors (Timoshenko *et al*, 2003). We had also shown that EP4 receptors, which are coupled to Gs proteins, are functional in MDA-MB-231 cells, contributing to their migration in response to endogenous PGE_2_ (Timoshenko *et al*, 2003). EP1 receptors are coupled with Gq proteins and typically they activate Ca^2+^-dependent intracellular signalling cascades. We demonstrated the functionality of EP1 receptors in MDA-MB-231 cells from a transient and moderate increase in intracellular Ca^2+^ levels in response to exogenous PGE_2_ (10 *μ*M) and an EP1 receptor agonist 17-phenyl trinor PGE_2_ (10 *μ*M) (not shown).

**Figure 6:**
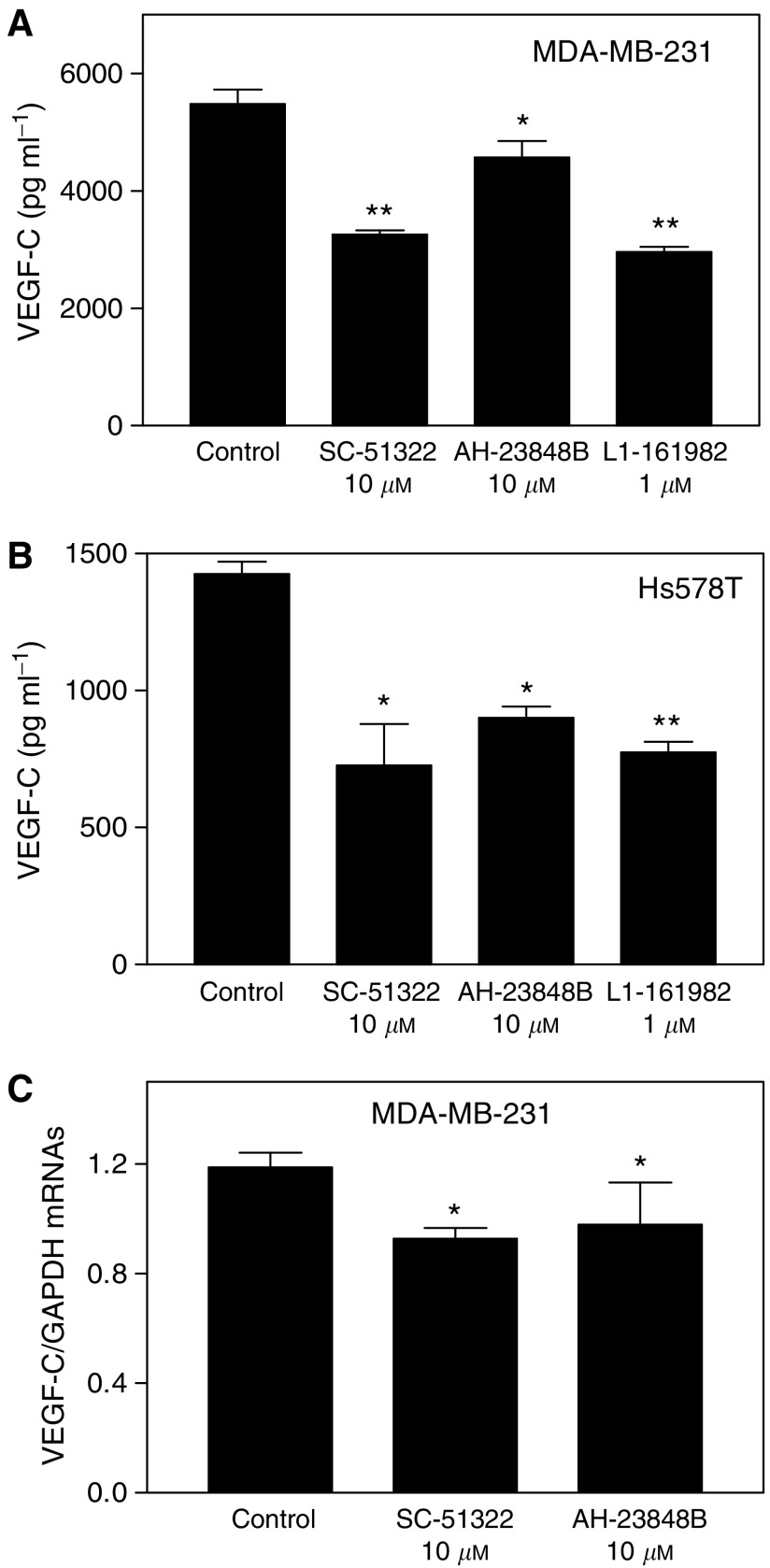
Roles of EP receptors in the regulation of VEGF-C production and expression by COX-2 expressing human breast cancer cells. Cells were treated for 24 h with antagonists of EP1 receptors (SC-51322) and EP4 receptors (AH-23848B and L-161982) in serum-free DMEM. (**A** and **B**) Effects of EP receptor antagonists on VEGF-C accumulation in culture media of MDA-MB-231 cells (**A**) and Hs538T cells (**B**). A consistent and significant inhibition of VEGF-C production was observed with highly specific EP1 receptor antagonist SC-51322 and EP4 receptor antagonist L-161982. (**C**) Quantification of VEGF-C mRNA levels in MDA-MD-231 cells after the 24 h exposure to EP receptor antagonists. Data represent mean±s.d. (*n*=4). ^*^P<0.05, ^**^P<0.01.

To test the role of individual EP receptors in an autocrine, PGE_2_-mediated regulation of VEGF-C synthesis, we treated COX-2-expressing Hs578 T and MDA-MB-231 cells with several EP antagonists including SC-51322, AH-23848B, and L-161982 at nontoxic final concentrations (10, 10 and 1 *μ*M, respectively) having no effect on cell proliferation/survival (data not shown) but earlier shown to block receptor activity (Coleman *et al*, 1994a, 1994b; Breyer *et al*, 2001; Tomita *et al*, 2002; Timoshenko *et al*, 2003). A strong inhibition of VEGF-C secretion was found with the selective EP1 receptor antagonist SC-51322 for both cell lines (Figure 6A and B). Two EP4 receptor antagonists, AH-23848B and L-161982, also variably inhibited VEGF-C secretion, however, the effects of L-161982 were stronger than those of AH-23848B. This difference is explained by a higher specificity and affinity of the former compound for EP4 receptors than the latter, which has a crossreactivity with TP receptors (Coleman *et al*, 1994a, 1994b). Inhibition of VEGF-C secretion in the presence of EP1/EP4 antagonists was at least partially due to downregulation of VEGF-C gene expression as demonstrated by real-time qPCR with MDA-MB-231 cells (Figure 6C). Possible role of EP2 and EP3 receptors could not be tested due to nonavailability of highly selective antagonists.

### VEGF-C synthesis by MDA-MB-231 cells is inhibited by inhibitors of Her-2/neu, Src and p38 MAP kinases

**Figure 7:**
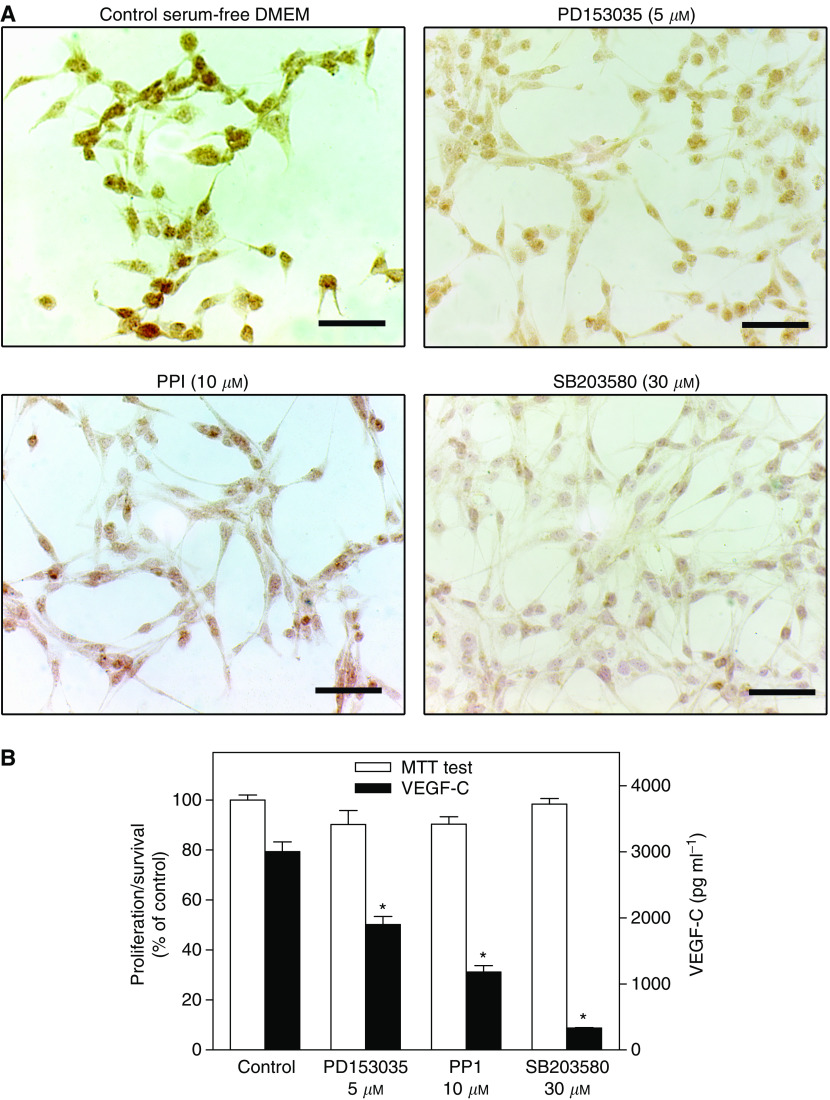
Reduction of VEGF-C expression in MDA-MB-231 cells induced by kinase inhibitors for Her2/Neu (PD153035), Src (PP1) and p38 MAPK (SB203580). MDA-MB-231 cells were cultured for 24 h in serum-free DMEM supplemented with 0.2 % BSA. (**A**) imunohistochemistry; all the kinase inhibitors at the concentrations employed inhibited cytoplasmic immunostaining for VEGF-C. Scale bar represents 60 *μ*m. (**B**) cell proliferation/survival (MTT test) and VEGF-C secretion; VEGF-C secretion, but not proliferation/survival, was inhibited by all the inhibitors. Data represent mean±s.d. (*n*=4), ^*^P<0.001.

VEGF-C synthesis in other cell types was reported to utilize signalling pathways associated with Her2/neu, Src, and p38 MAP kinases (Tsai *et al*, 2003; Su *et al*, 2004). We tested whether the application of the respective kinase inhibitors affected VEGF-C synthesis by MDA-MB-231 breast cancer cells. Cells were treated with PD153035 (Her2/neu kinase inhibitor; 5 *μ*M), PP1 (Src kinase inhibitor, 10 *μ*M) and SB203580 (p38 MAP kinase inhibitor, 30 *μ*M). As shown in Figure 7, all these inhibitors at nontoxic concentrations (having no effect on cell proliferation/survival as revealed by the MTT assay) inhibited VEGF-C secretion as well as the level of immunostaining for cytoplasmic VEGF-C production.

## DISCUSSION

The present study demonstrates for the first time that mRNA levels of COX-2, a well recognized functional marker for tumour progression, are highly correlated with VEGF-C mRNA levels in human breast cancer tissues and VEGF-C gene expression or secretion by breast cancer cell lines; that COX-2 or VEGF-C mRNA expression levels in breast cancer tissues are correlated with the expression of LYVE-1, a marker for lymphangiogenesis; that VEGF-C synthesis in breast cancer cells is stimulated, at least in part, by COX-2, EP1 and EP4 receptor activity.

The stimulatory role of COX-2 in breast cancer progression has earlier been explained by multiple PGE_2_-dependent mechanisms: an inactivation of antitumour immune cells (Lala and Saarloos, 1994), a stimulation of cancer cell growth and survival (Basu *et al*, 2005), migration (Rozic *et al*, 2001; Timoshenko *et al*, 2003), invasiveness (Rozic *et al*, 2001) and angiogenesis (Lala *et al*, 1997; Rozic *et al*, 2001). Present study demonstrates an additional role of COX-2-in human breast cancer: a stimulation of VEGF-C and thereby lymphangiogenesis *in situ*, also reported recently for non-small cell lung cancer cells (Su *et al*, 2004). The role of lymphangiogenesis for lymphatic metastases in human breast cancer patients remains a controversial issue. Intratumoural lymphangiogenesis is a feature of inflammatory breast cancer (Van der Auwera *et al*, 2004), whereas peritumoral but not intratumoral lymphatics were demonstrated in invasive, noninflammatory breast cancer (Vleugel *et al*, 2004). In the present study, which measured LYVE-1 mRNA levels in noninflammatory breast cancer tissues as indicators of lymphangiogenesis, the location of the lymphatics remains undetermined. *In situ* hybridization and immunostaining on a larger number of samples remain as future goals to resolve this issue. It is interesting to note that VEGF-C immunostaining in breast cancer tissues was reported to show a significant correlation with tumour cell invasion of lymphatic vessels at the microscopic level, but not with lymph node metastasis in one study (Kinoshita *et al*, 2001), whereas another study using a larger sample size including a higher proportion of VEGF-C expressing specimens demonstrated a positive correlation with nodal metastasis (Nakamura *et al*, 2003). Further studies are needed to examine whether lymphangiogenic role of VEGF-C is essential for lymphatic metastasis of breast cancer. Nevertheless, VEGF-C overexpression can now be added to the list of biomarkers such as overexpression of COX-2, Her-2/neu and VEGF-A which indicate poor prognosis in breast cancer patients (Zhang *et al*, 2003).

A strong positive association between COX-2 and VEGF-C expression noted here in breast cancer cell lines as well as in breast cancer tissues would suggest that breast cancer cells within the lesions served as the source of COX-2 or VEGF-C. However, we have not excluded the possibility that stromal cells and/or immigrant leukocytes may also be the source of both molecules. COX-2 mRNA expression has also been positively correlated with VEGF-A mRNA expression in human breast cancer specimens (Kirkpatrick *et al*, 2002), however, this association is much weaker than that we have noted with VEGF-C (correlation coefficients 0.55 *vs* 0.94). An association between COX-2 and VEGF-C, either at the mRNA or protein levels, has also been reported for squamous cell carcinomas of the head and neck (Kyzas *et al*, 2005), oesophagus (Byeon *et al*, 2004; von Rahden *et al*, 2005), and non-small cell lung cancer (Su *et al*, 2004). This relationship can be explained in two ways: that both genes are upregulated by a common factor, or that one upregulates the other. The first explanation derives support from the facts that certain growth factors and inflammatory cytokines (such as IL-1*β*, TNF*α*, PDGF, TGF*β* and heregulin-*β*1) can stimulate VEGF-C mRNA expression or protein synthesis in certain cells (Enholm *et al*, 1997; Ristimäki *et al*, 1998; Tsai *et al*, 2003), and that they can also upregulate COX-2 which is a cytokine-responsive gene (Ristimäki *et al*, 1994). We have not excluded this possibility *in situ*. The second explanation, that is, COX-2-mediated upregulation of VEGF-C has been validated in the present study using breast cancer cell lines and was also reported with cell lines derived from non-small cell lung cancer (Su *et al*, 2004) as well as oesophageal adenocarcinoma (von Rahden *et al*, 2005). However, our data show that COX-2 is an important, but not the sole regulator of VEGF-C, since inhibition of COX-2 activity or a knock down of the COX-2 gene caused a moderate but not absolute suppression of VEGF-C expression and secretion. The existence of NF-*κ*B binding sites in the promoter regions of both genes (Appleby *et al*, 1994; Chilov *et al*, 1997) may suggest additional intrinsic mediator(s) causing a parallel upregulation of both genes via NF-*κ*B pathway.

We have shown that COX-2-mediated upregulation of VEGF-C is, at least in part, dependent on endogenous PGE_2_-mediated signalling via EP1 and EP4 receptors. EP1 activation was also reported to contribute to VEGF-C upregulation in non-small cell lung cancer cells (Su *et al*, 2004). We had earlier reported the contribution of EP4 in endogenous PGE_2_-stimulated migration of MDA-MB-231 cells (Timoshenko *et al*, 2003), but did not exclude the role of EP1 in this process. EP2 has recently been implicated in COX-2-mediated mammary hyperplasia (Chang *et al*, 2005). Taken together, these results reveal that EP1, EP2 and EP4 receptors contribute to breast cancer progression, similar to their documented roles in experimental colon carcinogenesis (Hull *et al*, 2004).

Downstream signalling molecules responsible for EP1- or EP4-mediated VEGF-C upregulation in breast cancer remain to be identified. The promoter region of VEGF-C gene contains putative binding sites for Sp1, AP-2 and NF-*κ*B (Chilov *et al*, 1997) and, therefore, activation of any of these transcription factors may be instrumental in upregulation of VEGF-C. VEGF-C upregulation in case of non-small cell lung cancer cells was shown to follow EP1-mediated transactivation of Her-2/neu via Src kinase pathway (Su *et al*, 2004). In turn, Src kinase pathway, in some systems, was reported to cause activation of NF-*κ*B (Courter *et al*, 2005) or Sp1 (Xu *et al*, 2004). Furthemore, Her-2/neu kinase stimulation by heregulin-*β*1 was shown to upregulate VEGF-C in COX-2 negative MCF-7 cells following activation of p38 MAP kinase and NF-*κ*B (Tsai *et al*, 2003). In support of some of these findings, we have shown here that VEGF-C synthesis by COX-2 expressing MDA-MB-231 breast cancer cells was dependent on Her-2/neu, p38 MAP and Src kinases. Whether and how these pathways are triggered by activation of EP1 or EP4 in breast cancer cells remain to be examined. Such studies may highlight newer therapeutic targets for breast cancer in addition to COX-2, Her2/neu and EP receptors as revealed here.

## Figures and Tables

**Table 1:** Oligonucleotide Primer Pairs for RT–PCR

**Gene (accession #)**	**Forward primer sequence, 5′ → 3′ (positions)**	**Reverse primer sequence, 5′ → 3′ (positions)**	**Product size, bp**
VEGF-A	CTTGCCTTGCTGCTCTACC	CACACAGGATGGCTTGAAG	201
(NM_003376)	(1072–1090)	(1272–1254)	
VEGF-B	CCAGAGGAAAGTGGTGTCAT	AGTGGGATGGGTGATGTCAG	415
(NM_003377)	(142–161)	(556–535)	
VEGF-C	CGGGAGGTGTGTATAGATGTG	ATTGGCTGGGGAAGAGTTTG	583
(NM_005429)	(830–850)	(1412–1393)	
VEGF-D	CAGTGAAGCGATCATCTCAG	TACGAGGTGCTGGTGTTCATAC	397
(NM_004469)	(592–611)	(988–967)	
COX-2	GAATGGGGTGATGAGCAGTT	CAGAAGGGCAGGATACAGC	561
(NM_000963)	(1056–1075)	(1616–1598)	
LYVE-1	CCAGTGAGCCGACAGTTTGGAG	CAGGTATTGTAGAGTAAGGGGATGCC	184
(AF118108)	(445–466)	(628–603)	
GAPDH	ACCACAGTCCATGCCATCAC	TCCACCACCCTGTTGCTGTA	452
(AF261085)	(629–648)	(1080–1061)	
